# Supplementary material for: Inhibitory Effects of Nitrogenous Metabolites from a Marine-Derived Streptomyces bacillaris on Isocitrate Lyase of Candida albicans
Source: Mar Drugs. 2022 Feb 13;20(2):138. doi: 10.3390/md20020138 (PMC8878140; doi:10.3390/md20020138)
Supplement: Supplementary file 1 [file marinedrugs-20-00138-s001.zip › marinedrugs-1588795-supplementary.pdf]

Supporting Information

Inhibitory Effects of Nitrogenous Metabolites from a Marine-derived *Streptomyces bacillaris* on Isocitrate Lyase of *Candida albicans*

Beomkoo Chung [1], Ji-Yeon Hwang [2], Sung Chul Park [2], Oh-Seok Kwon [2], Eunji Cho [1], Jayho Lee [1], Hyi-Seung Lee [3], Dong-Chan Oh [2], Jongheon Shin [2,*] and Ki-Bong Oh [1,*]

[1] Department of Agricultural Biotechnology, College of Agriculture and Life Sciences, Seoul National University, Seoul 08826, Korea

[2] Natural Products Research Institute, College of Pharmacy, Seoul National University, Seoul 08826, Korea

[3] Marine Natural Products Chemistry Laboratory, Korea Institute of Ocean Science and Technology, Busan 49111, Korea

Contents

Figure S1. The ^{1}H NMR (800 MHz, DMSO-d_6) spectrum of **1** S3

Figure S2. The ^{13}C NMR (200 MHz, DMSO-d_6) spectrum of **1** S4

Figure S3. The COSY (500 MHz, DMSO-d_6) spectrum of **1** S5

Figure S4. The HSQC (800 MHz, DMSO-d_6) spectrum of **1** S6

Figure S5. The HMBC (800 MHz, DMSO-d_6) spectrum of **1** S7

Figure S6. The NOESY (600 MHz, DMSO-d_6) spectrum of **1** S8

Figure S7. The ^{1}H NMR (500 MHz, MeOH-d_4) spectrum of **1** S9

Figure S8. HR-ESI-MS data of **1** S10

Figure S9. The ^{1}H NMR (800 MHz, DMSO-d_6) spectrum of **1a** S11

Figure S10. The ^{13}C NMR (200 MHz, DMSO-d_6) spectrum of **1a** S12

Figure S11. The NOESY (800 MHz, DMSO-d_6) spectrum of **1a** S13

Figure S12. HR-ESI-MS data of **1a** S14

Figure S13. The ^{1}H NMR (600 MHz, DMSO-d_6) spectrum of **2** S15

Figure S14. The ^{13}C NMR (150 MHz, DMSO-d_6) spectrum of **2** S16

Figure S15. The COSY (600 MHz, DMSO-d_6) spectrum of **2** S17

Figure S16. The HSQC (600 MHz, DMSO-d_6) spectrum of **2** S18

Figure S17. The HMBC (600 MHz, DMSO-d_6) spectrum of **2** S19

Figure S18. HR-ESI-MS data of **2** S20

Figure S19. The ^{1}H NMR (800 MHz, DMSO-d_6) spectrum (δ_H 1.3 – 2.4 ppm) of **1** S21

Figure S20. The ^{1}H NMR (600 MHz, DMSO-d_6) spectrum (δ_H 2.7 – 3.8 ppm)of **2** S22

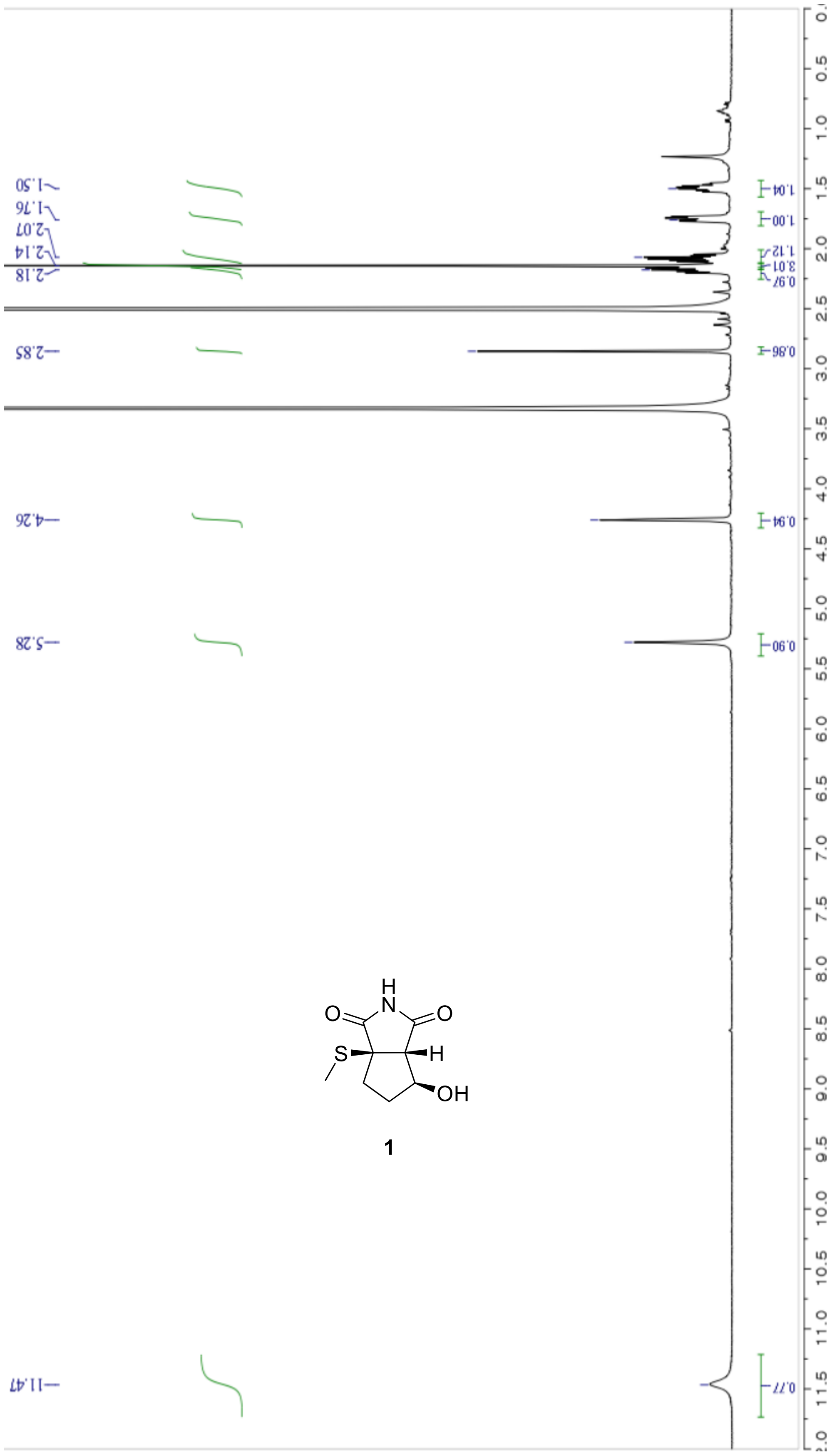

Figure S1. The ^{1}H NMR (800 MHz, DMSO-d_6) spectrum of **1.**

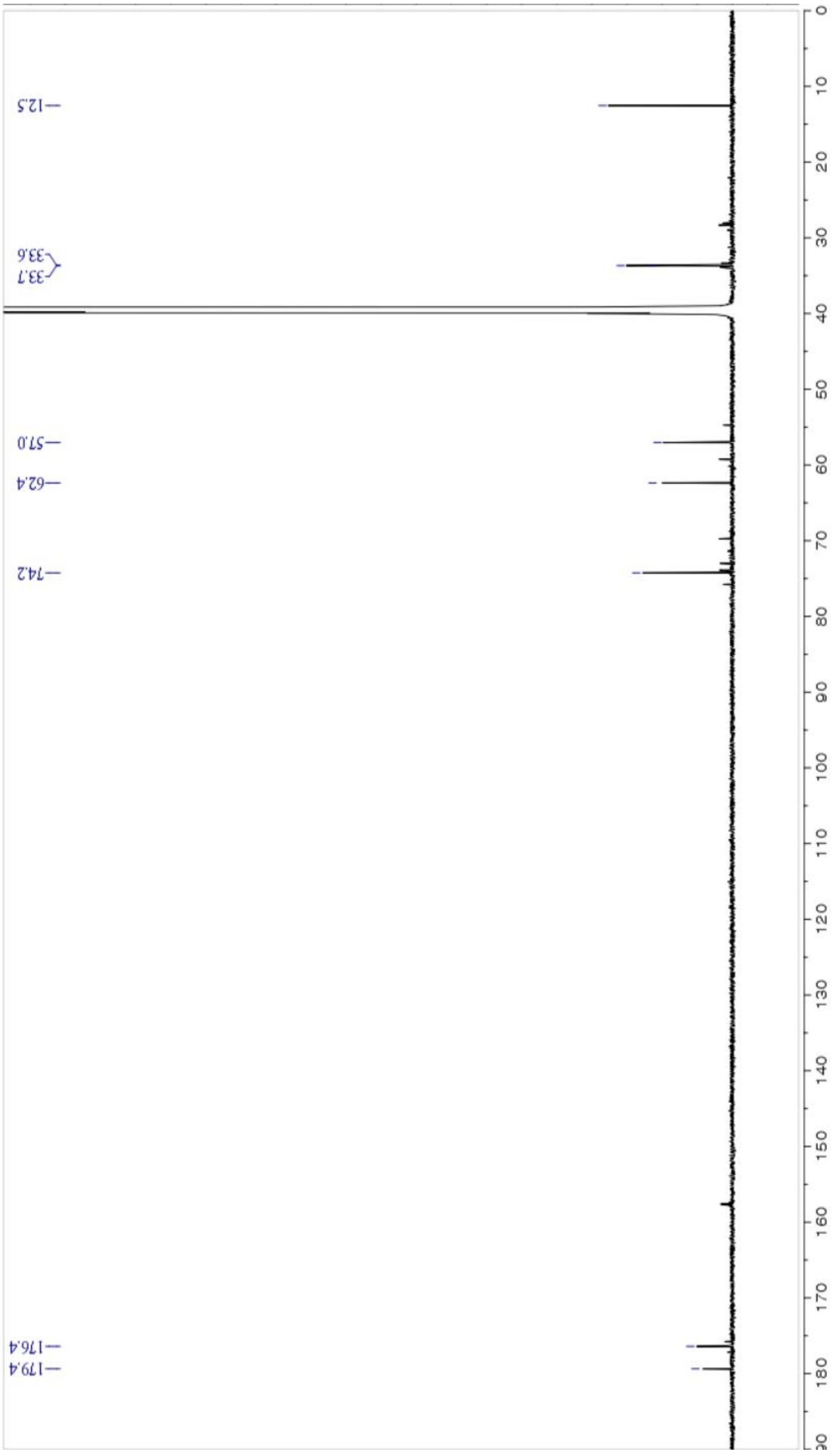

Figure S2. The ^{13}C NMR (200 MHz, DMSO-d_6) spectrum of **1**.

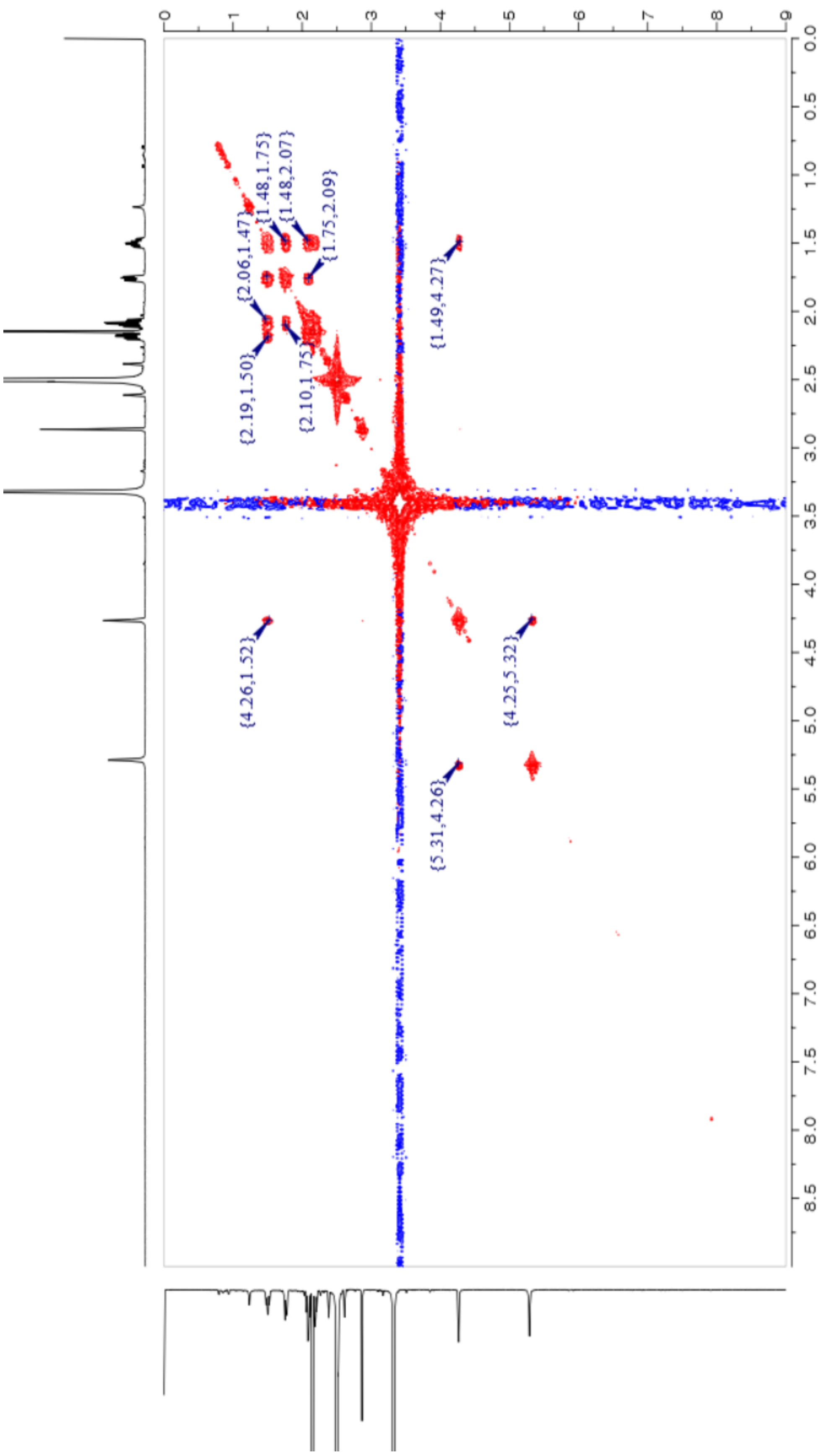

Figure S3. The COSY (500 MHz, DMSO-d_6) spectrum of **1**.

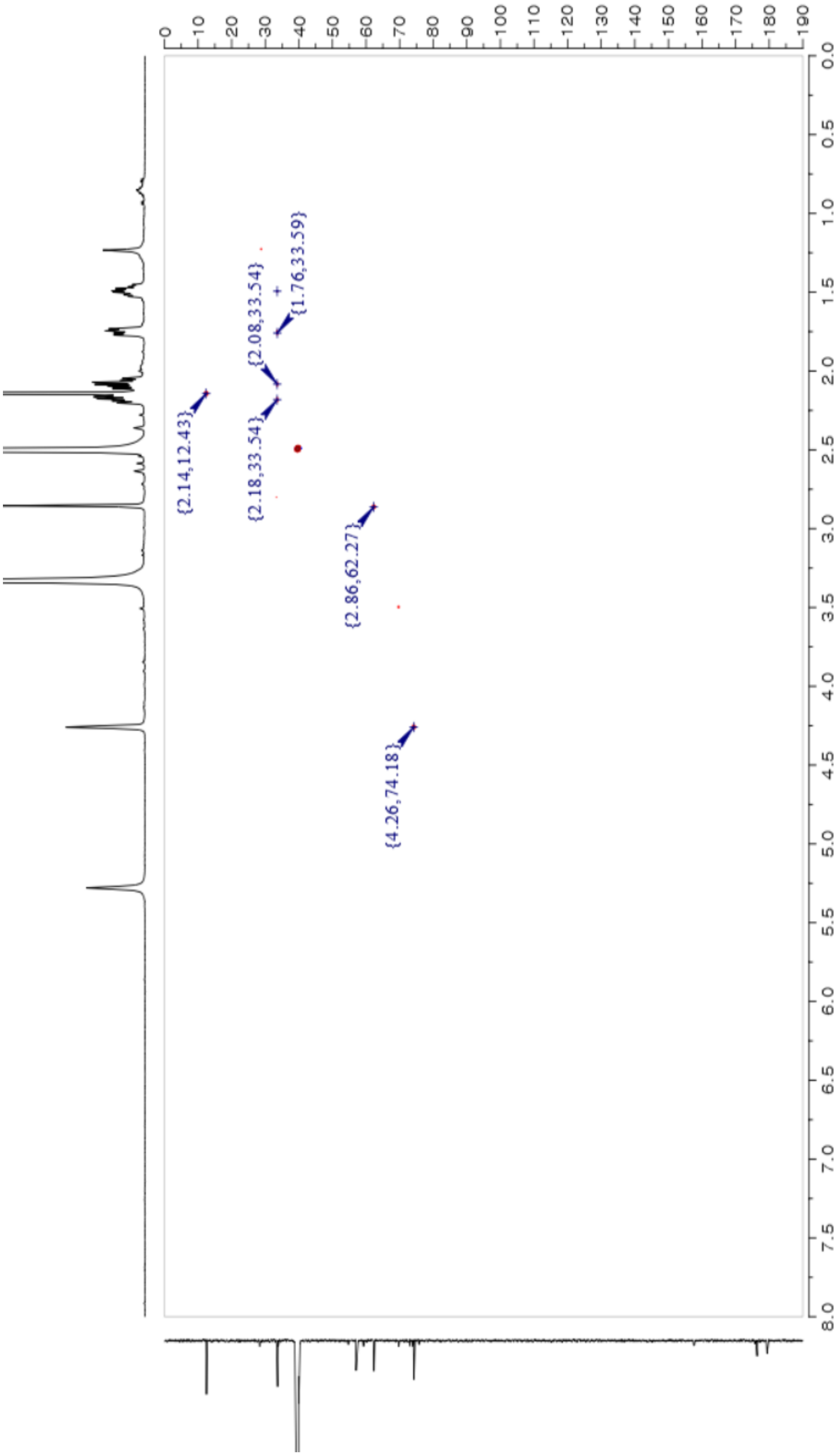

Figure S4. The HSQC (800 MHz, DMSO-d_6) spectrum of **1**.

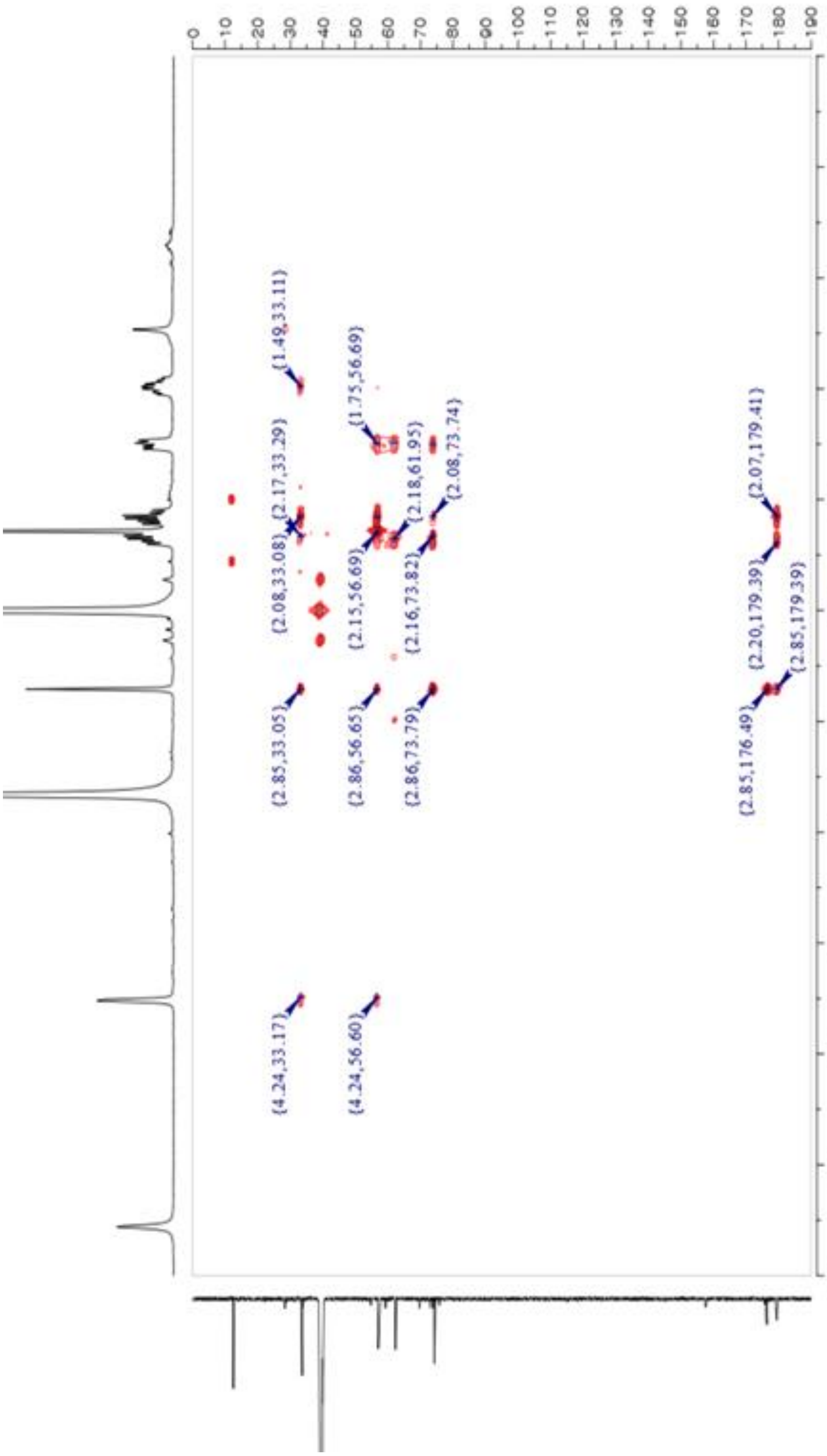

Figure S5. The HMBC (800 MHz, DMSO-d_6) spectrum of **1**.

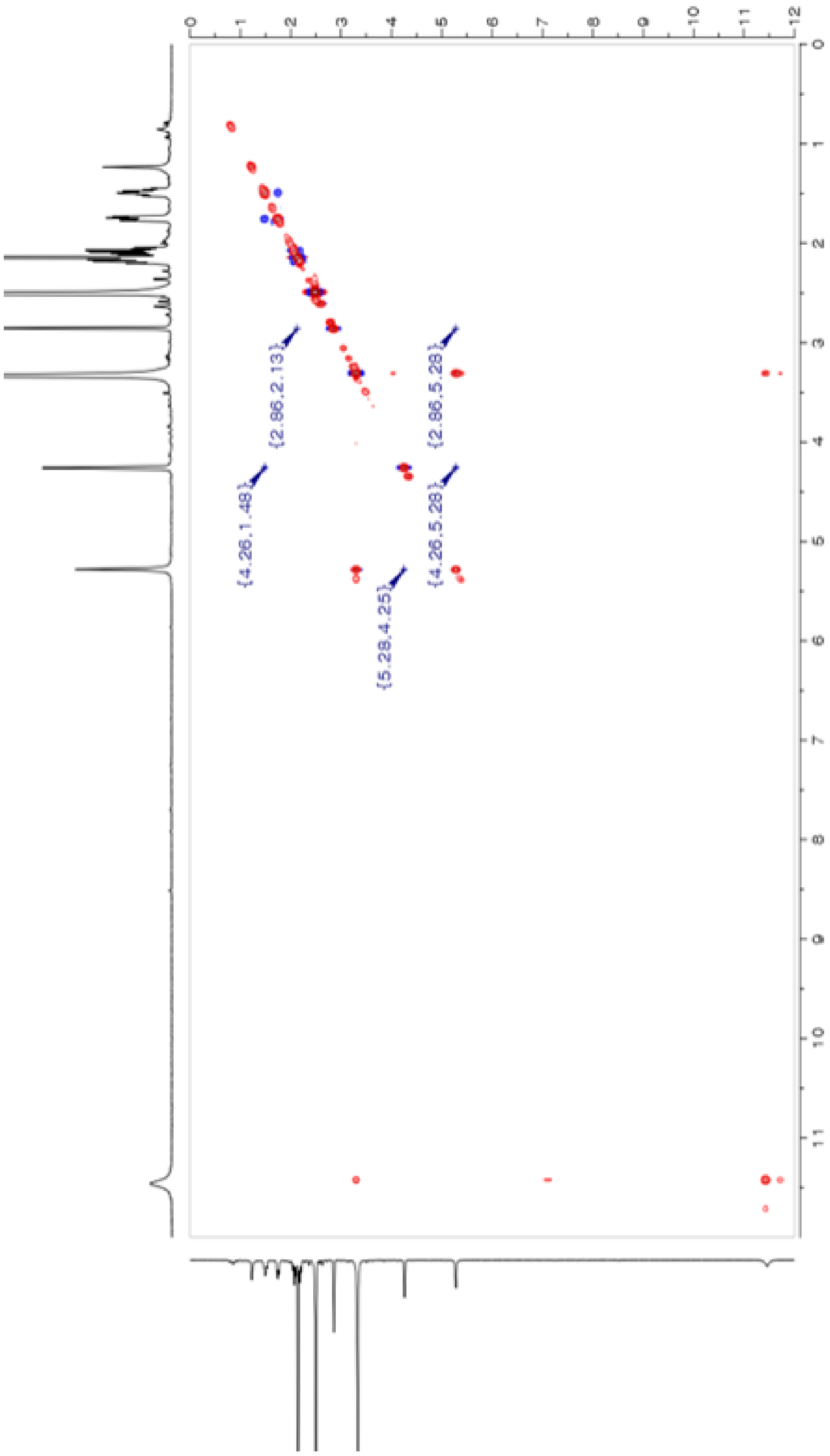

Figure S6. The NOESY (600 MHz, DMSO-d_6) spectrum of **1**.

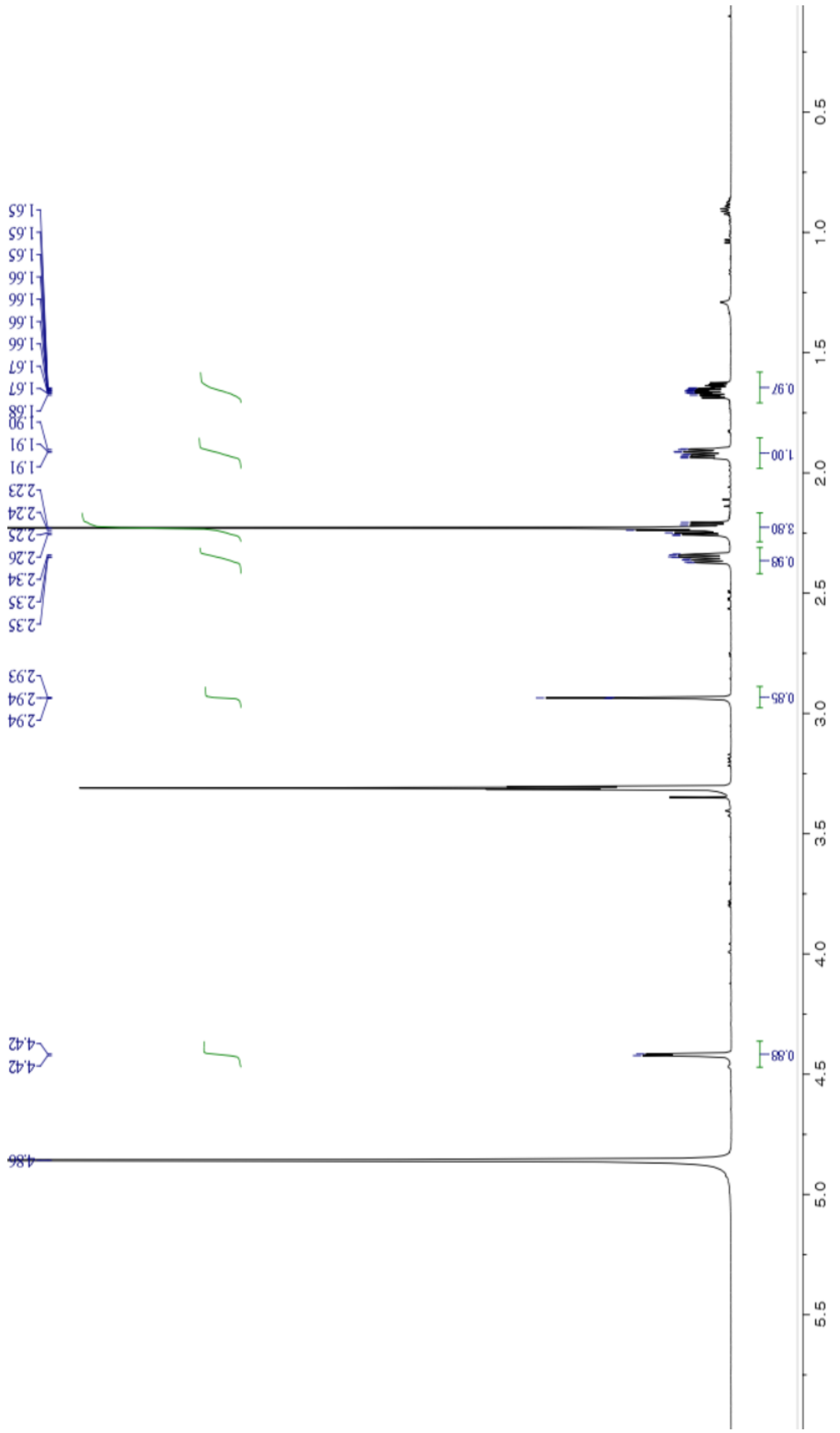

Figure S7. The ^{1}H NMR (500 MHz, MeOH-d_4) spectrum of **1**.

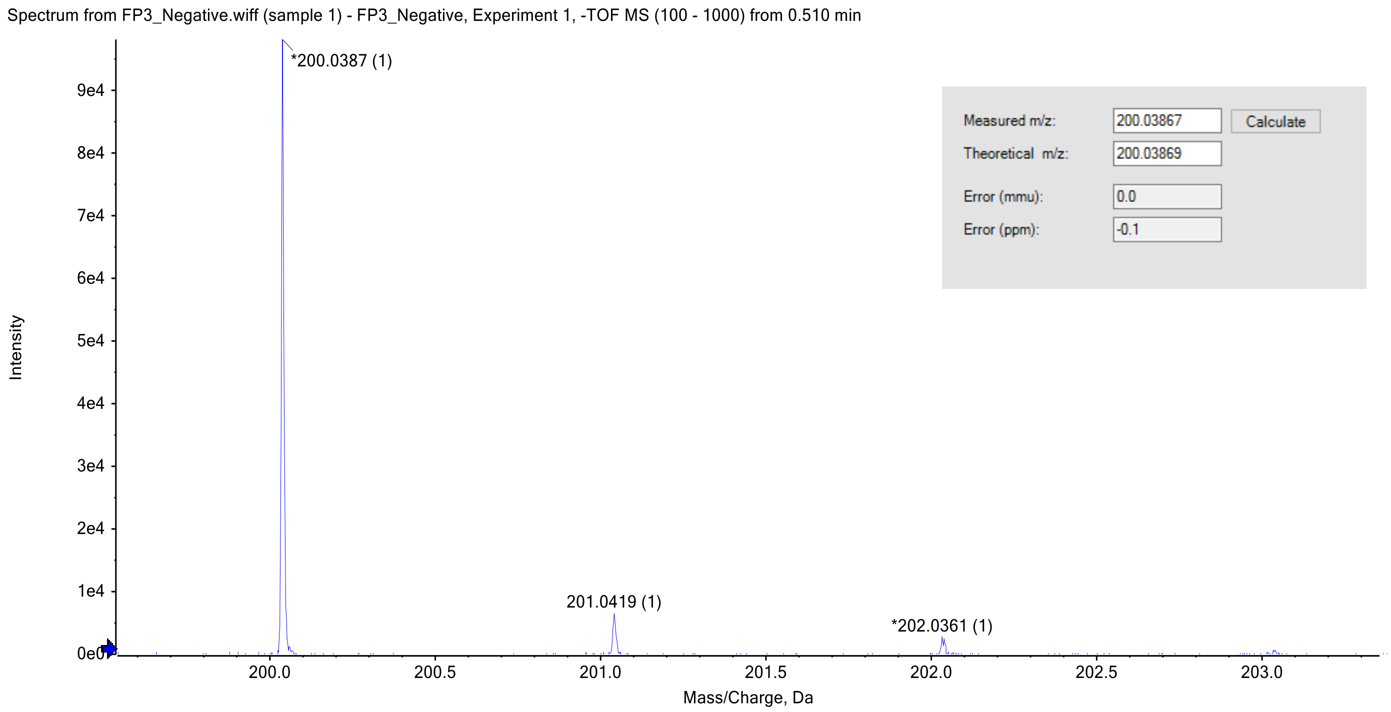

Figure S8. HR-ESI-MS data of **1**.

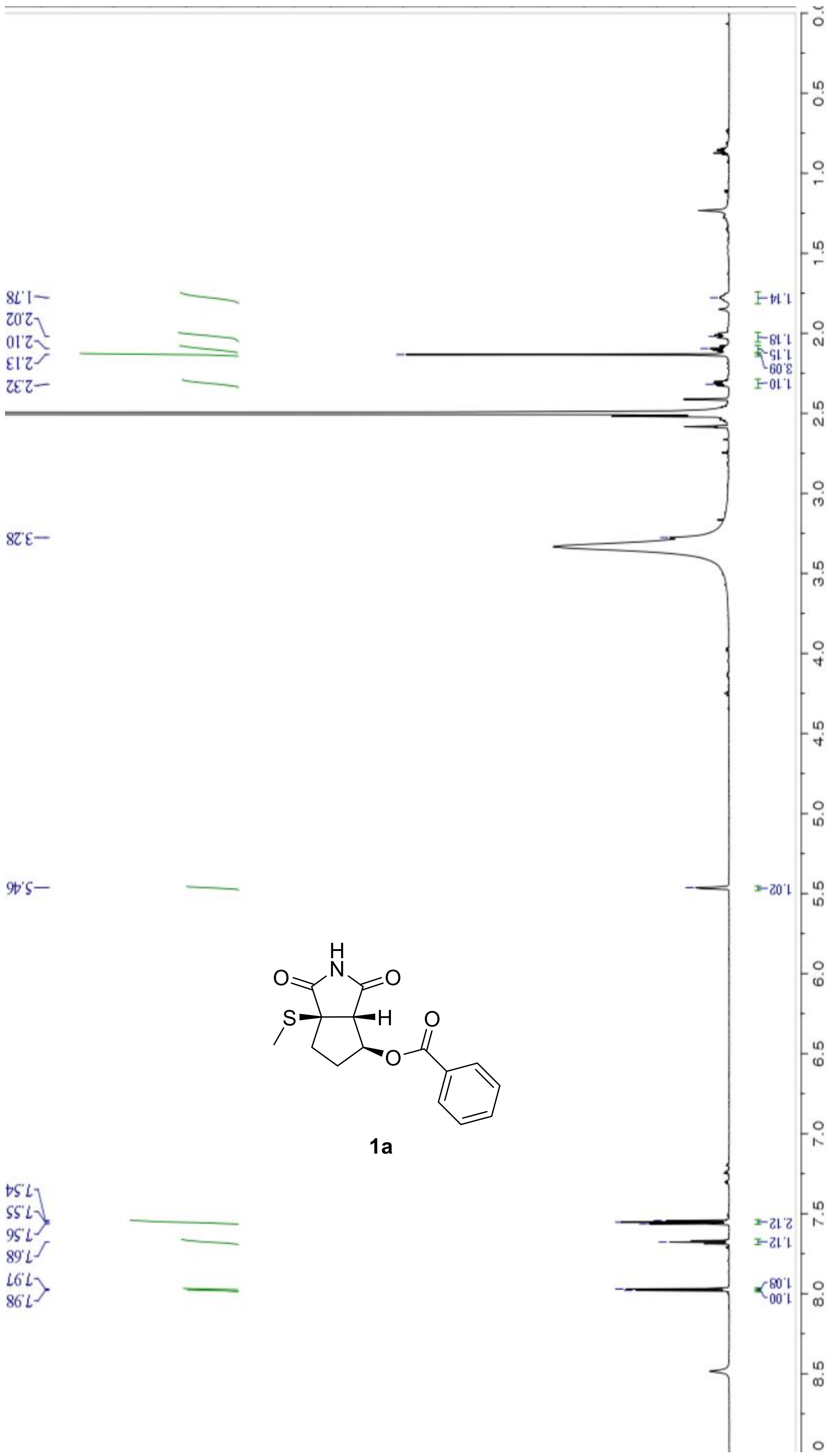

Figure S9. The ^{1}H NMR (800 MHz, DMSO-d_6) spectrum of **1a**.

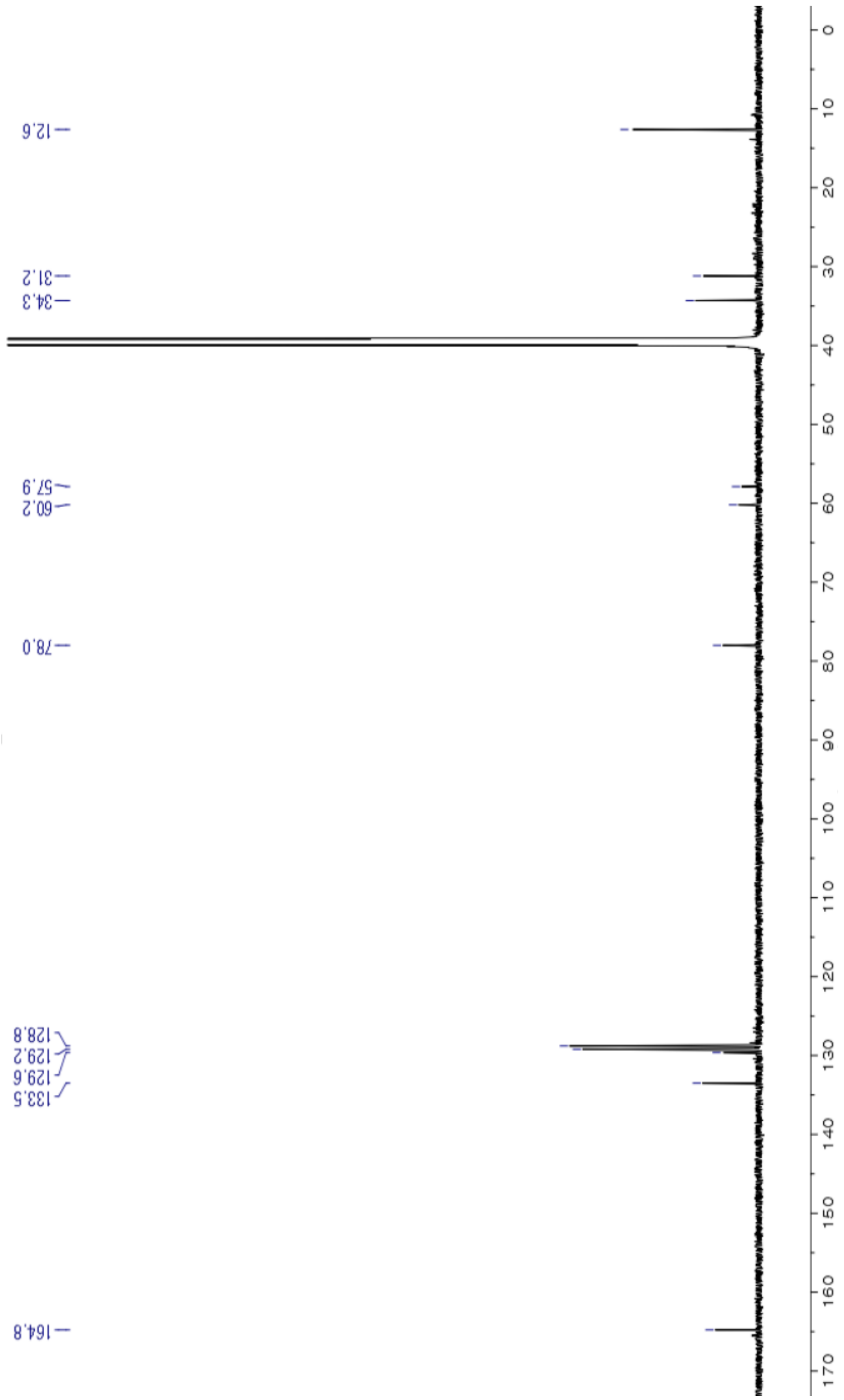

Figure S10. The ^{13}C NMR (200 MHz, DMSO-d_6) spectrum of **1a**.

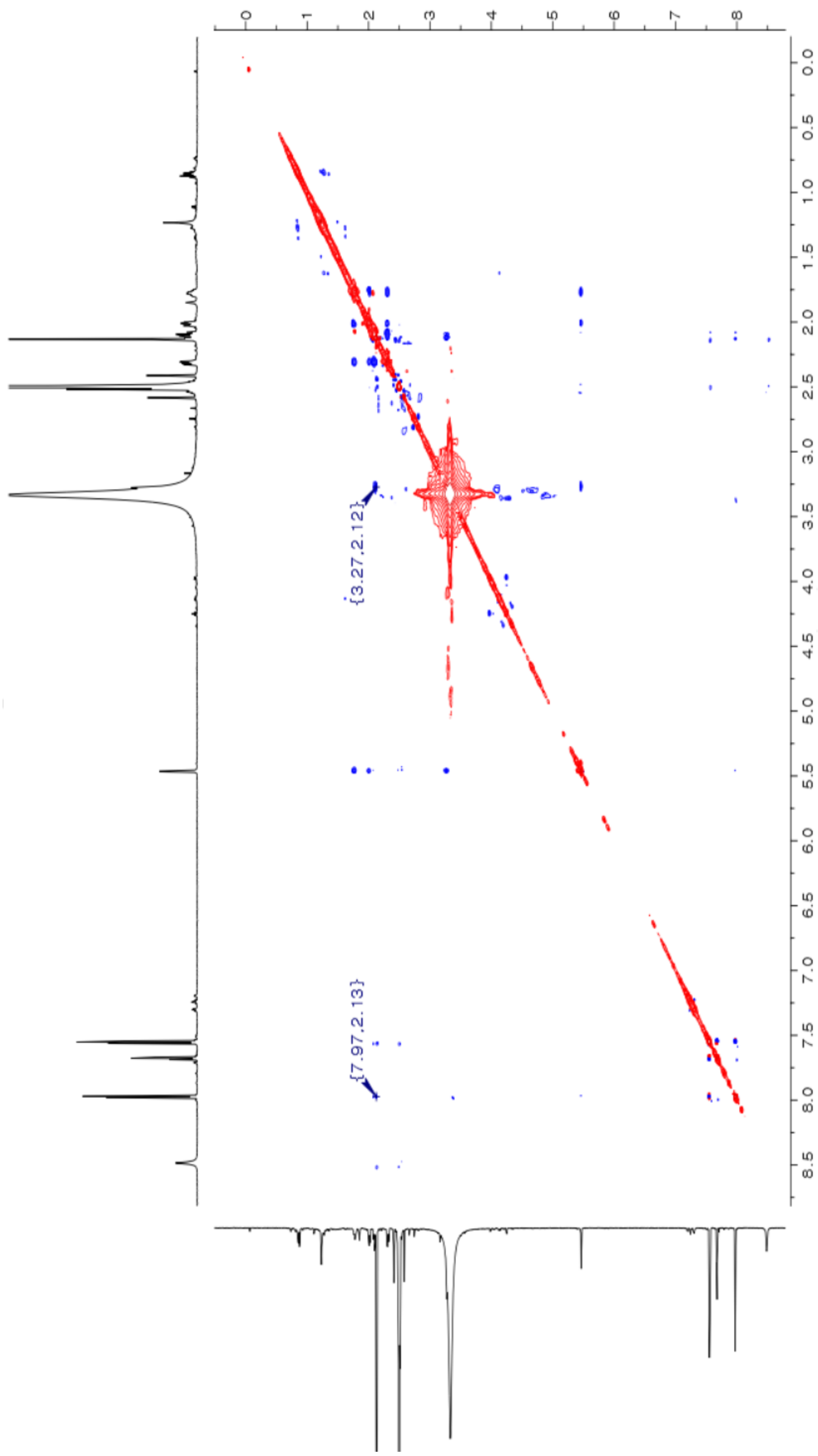

Figure S11. The NOESY (800 MHz, DMSO-d_6) spectrum of **1a**.

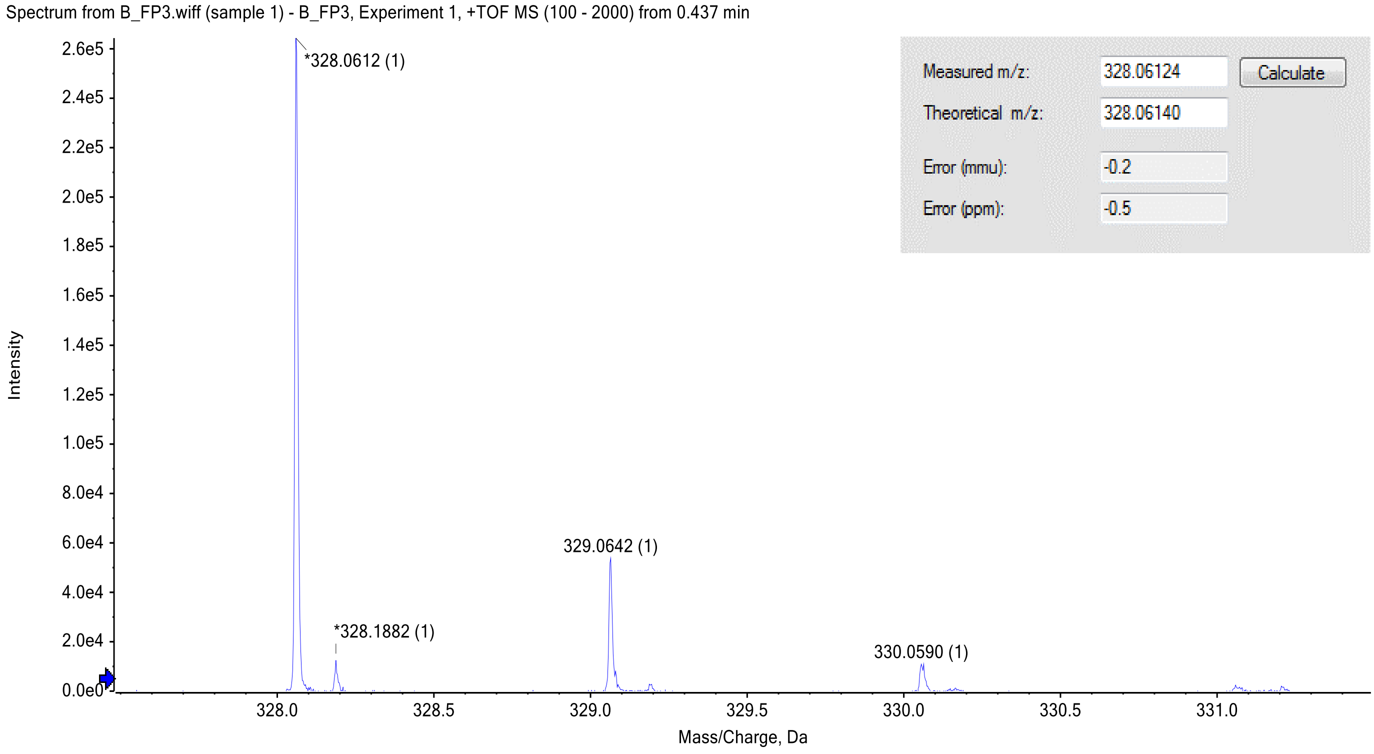

Figure S12. HR-ESI-MS data of **1a**.

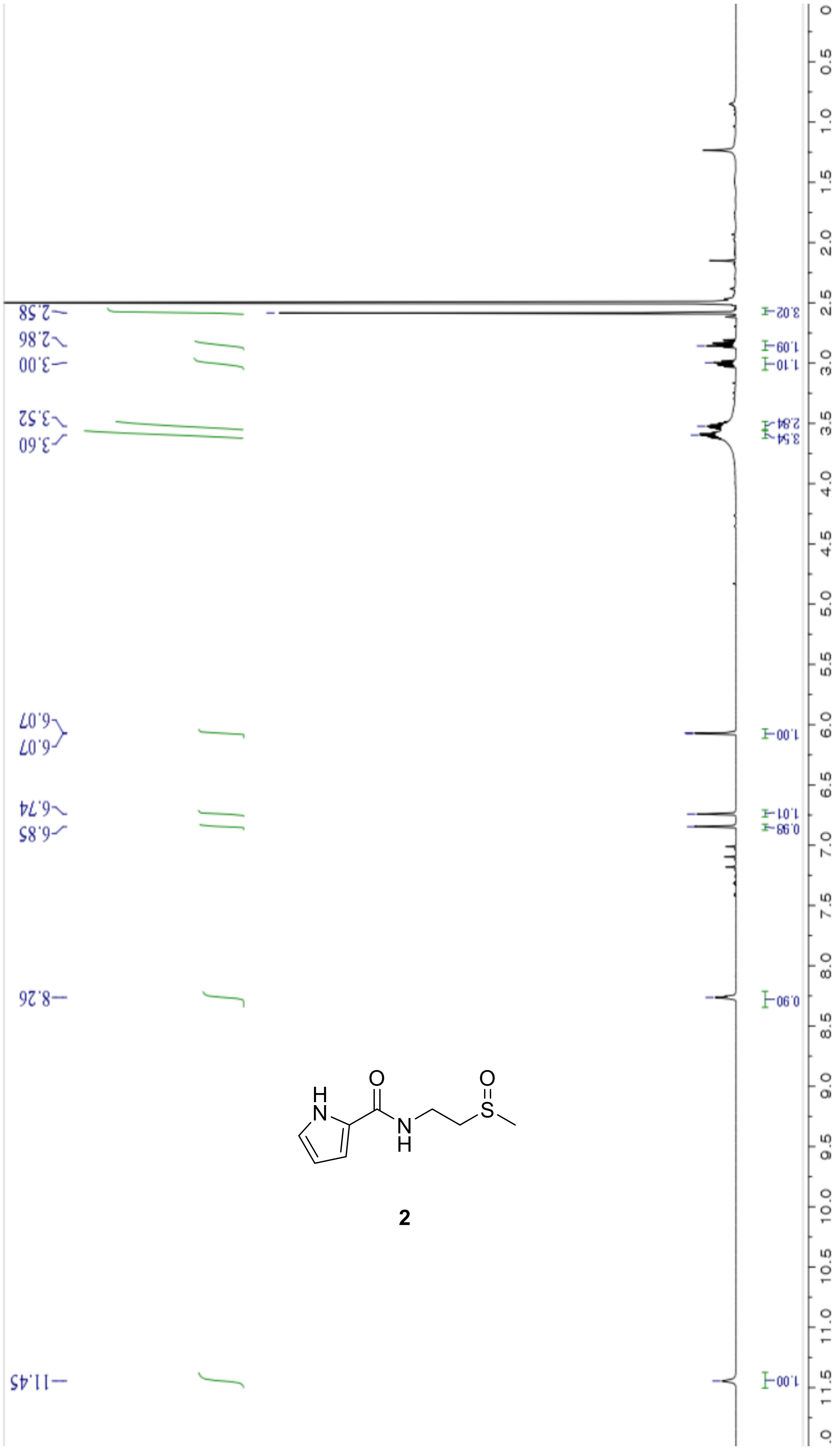

Figure S13. The ^{1}H NMR (600 MHz, DMSO-d_6) spectrum of **2**.

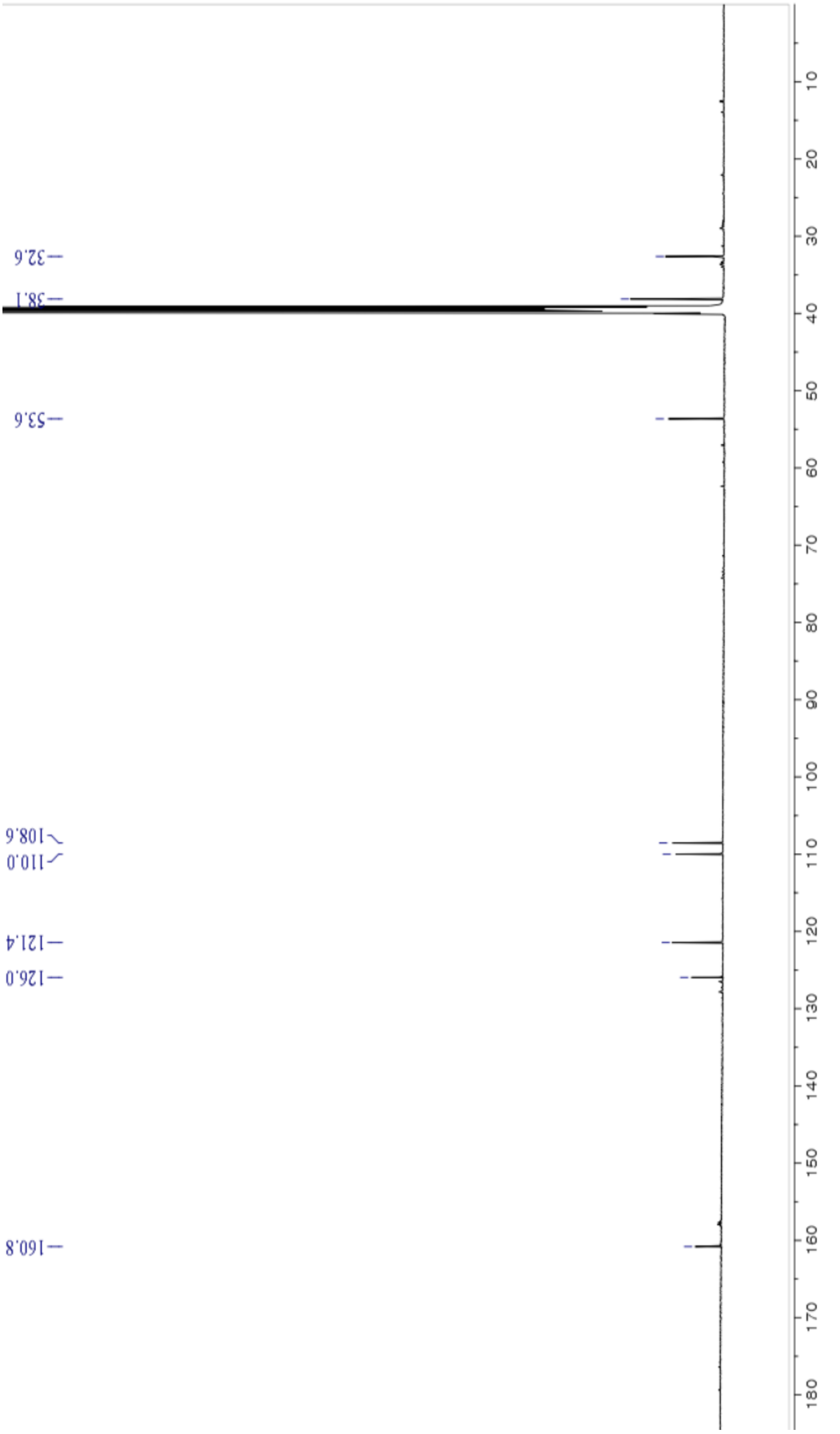

Figure S14. The ^{13}C NMR (150 MHz, DMSO-d_6) spectrum of **2**.

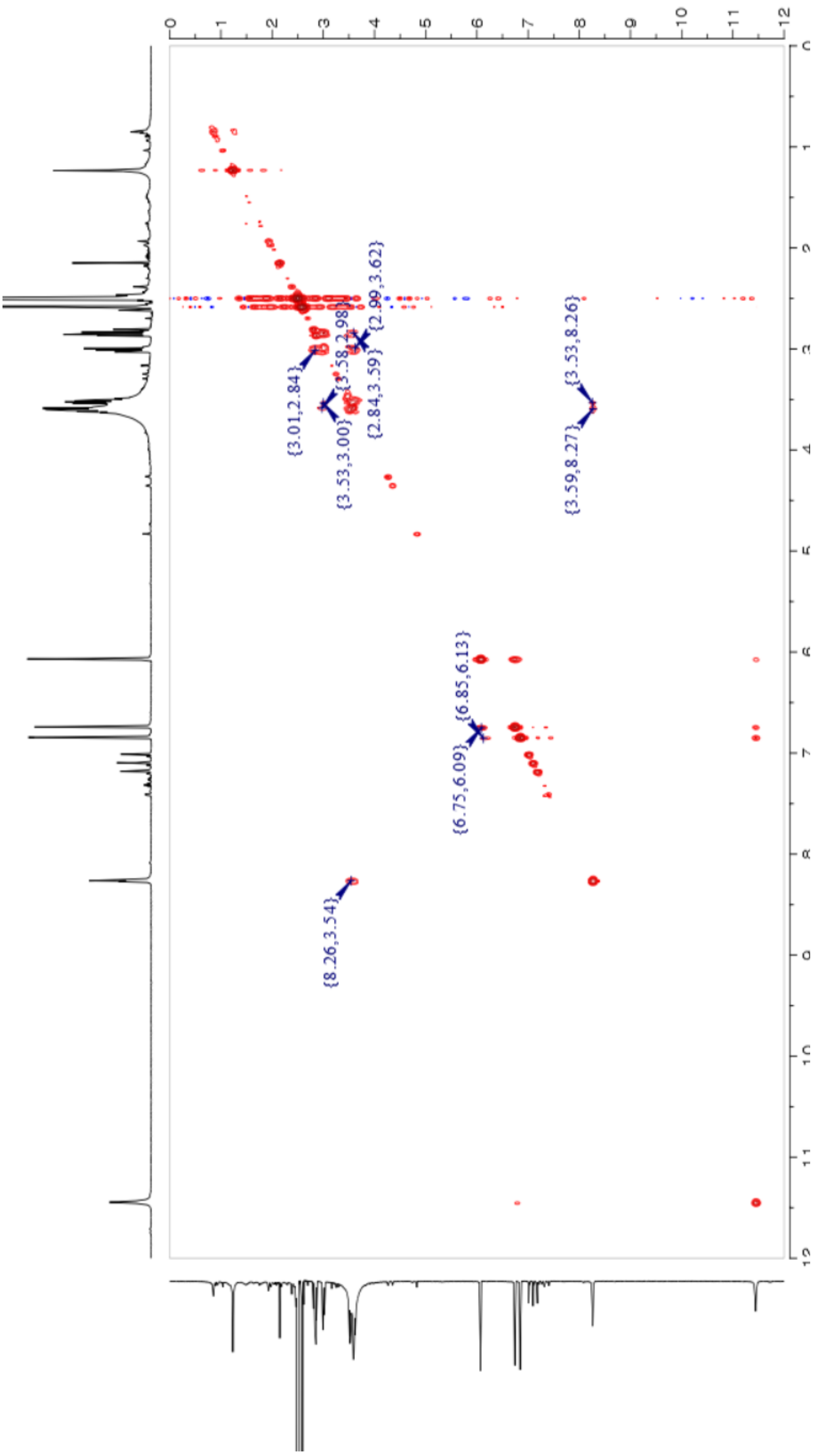

Figure S15. The COSY (600 MHz, DMSO-d_6) spectrum of **2**.

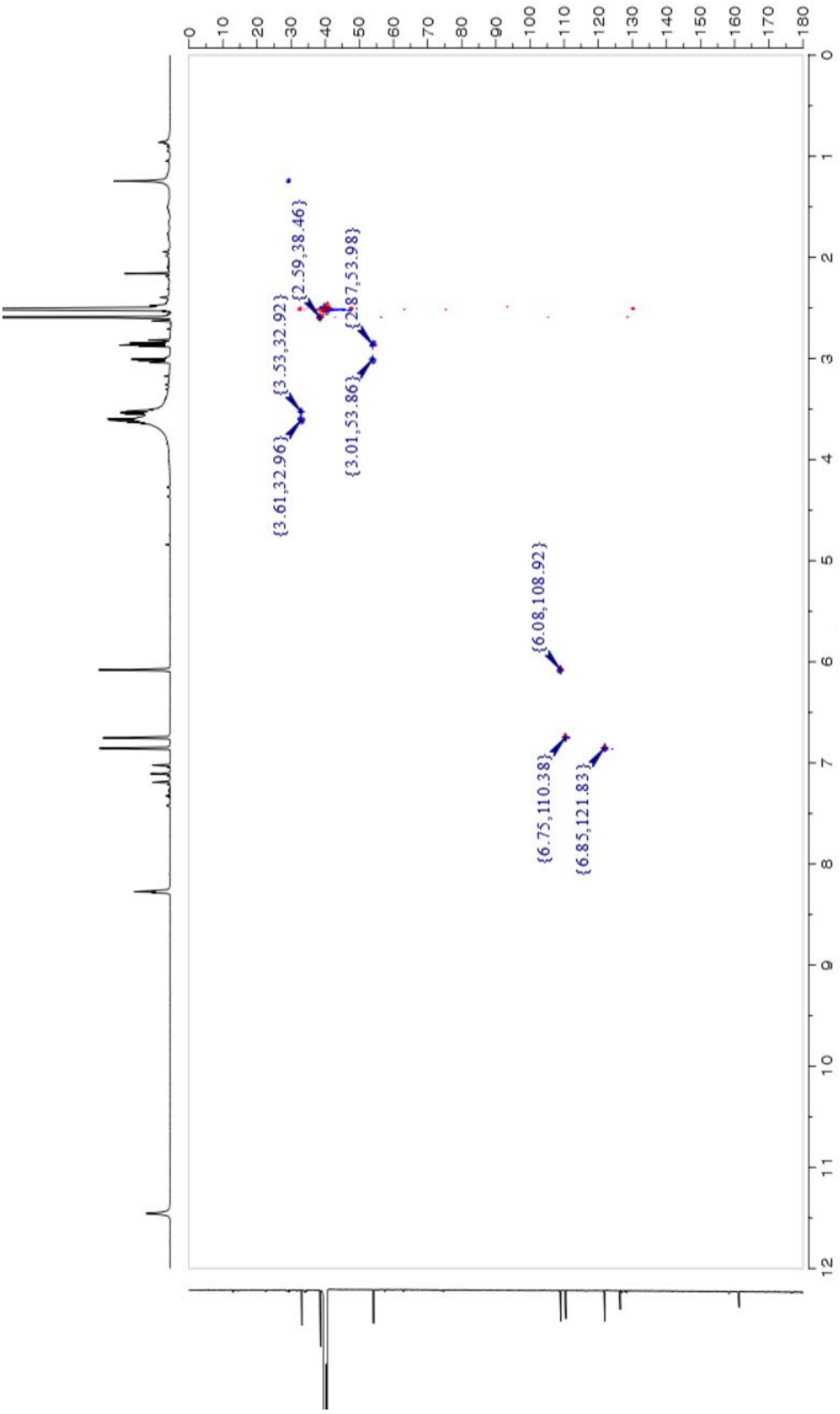

Figure S16. The HSQC (600 MHz, DMSO-d_6) spectrum of **2**.

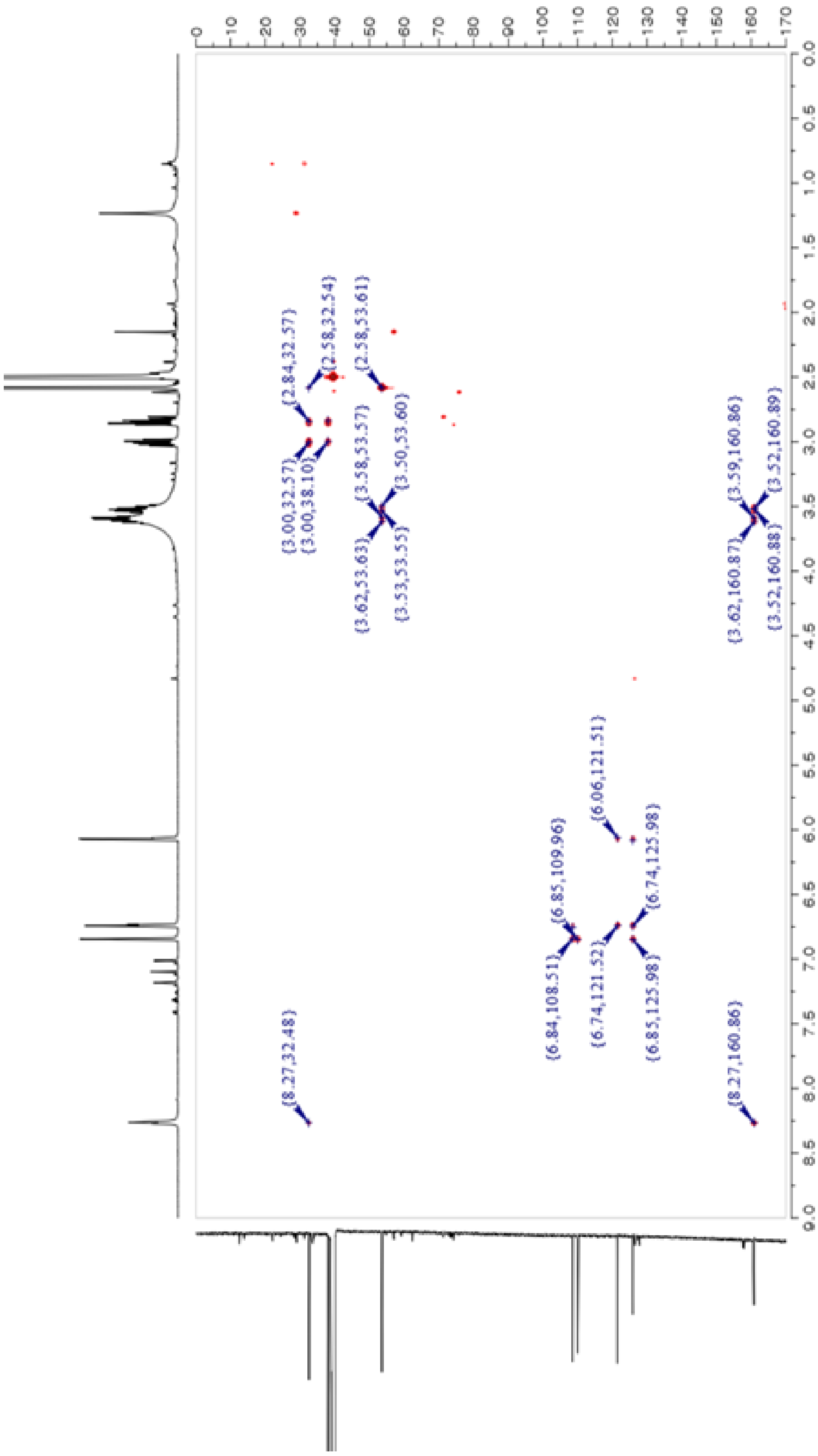

Figure S17. The HMBC (600 MHz, DMSO-d_6) spectrum of **2**.

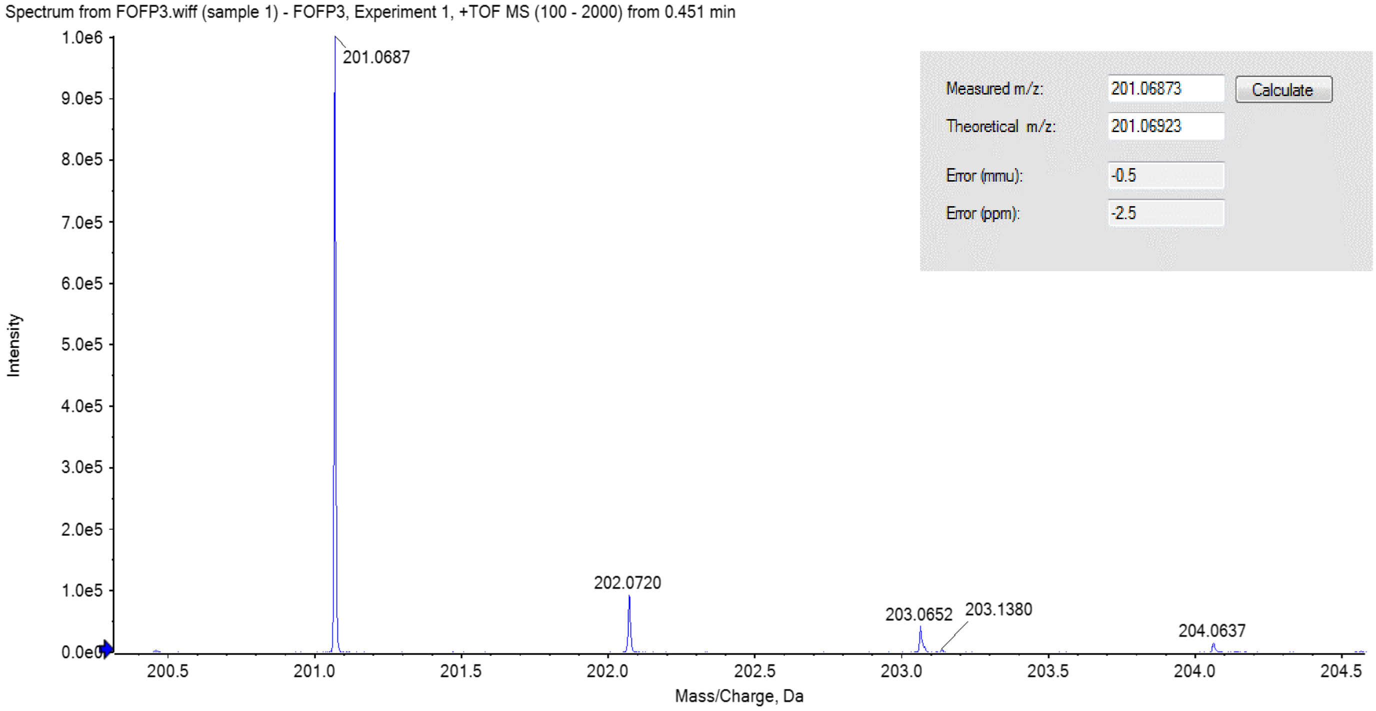

Figure S18. HR-ESI-MS data of **2**.

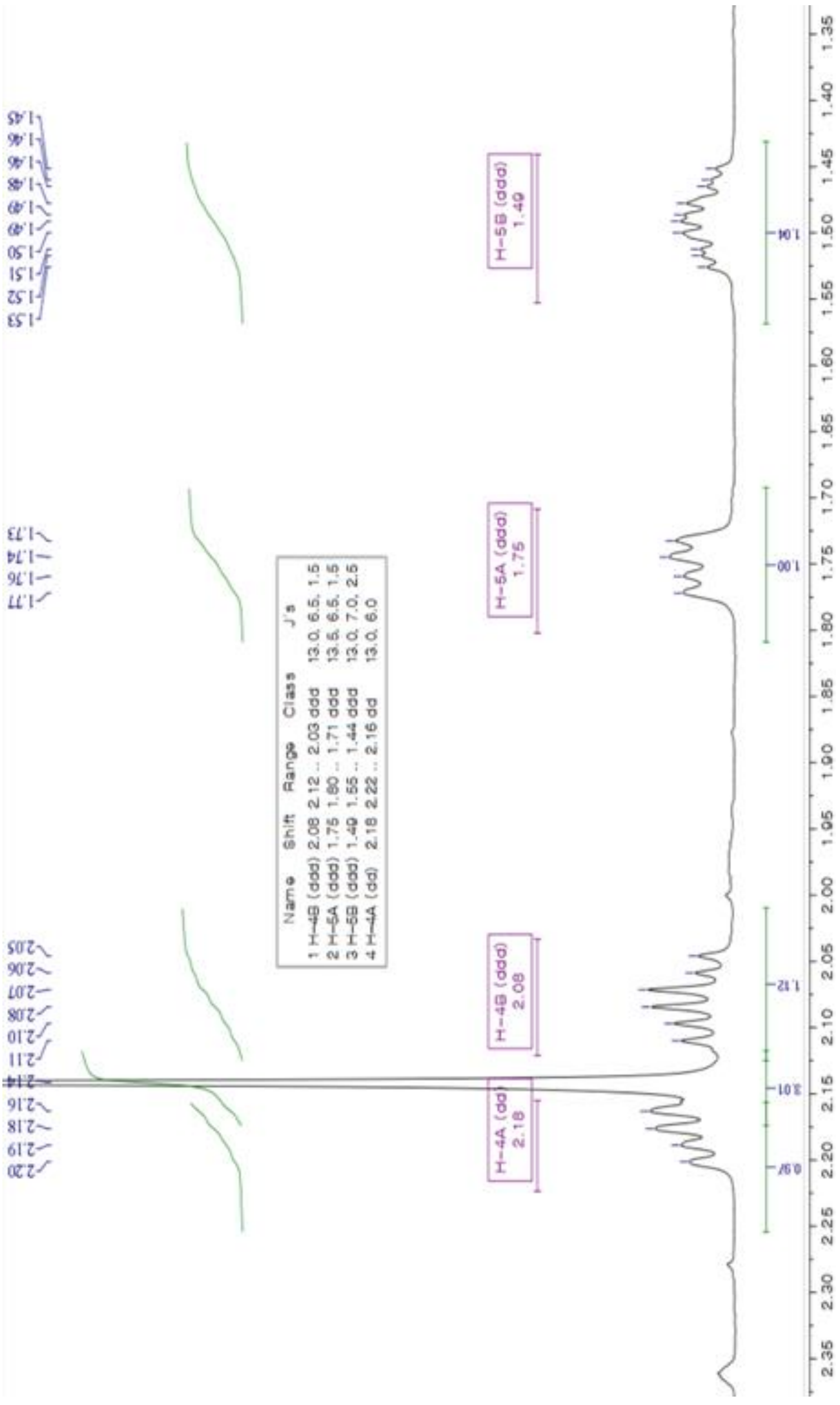

Figure S19. The ^{1}H NMR (800 MHz, DMSO-d_6) spectrum (δ_H 1.3 – 2.4 ppm) of **1**.

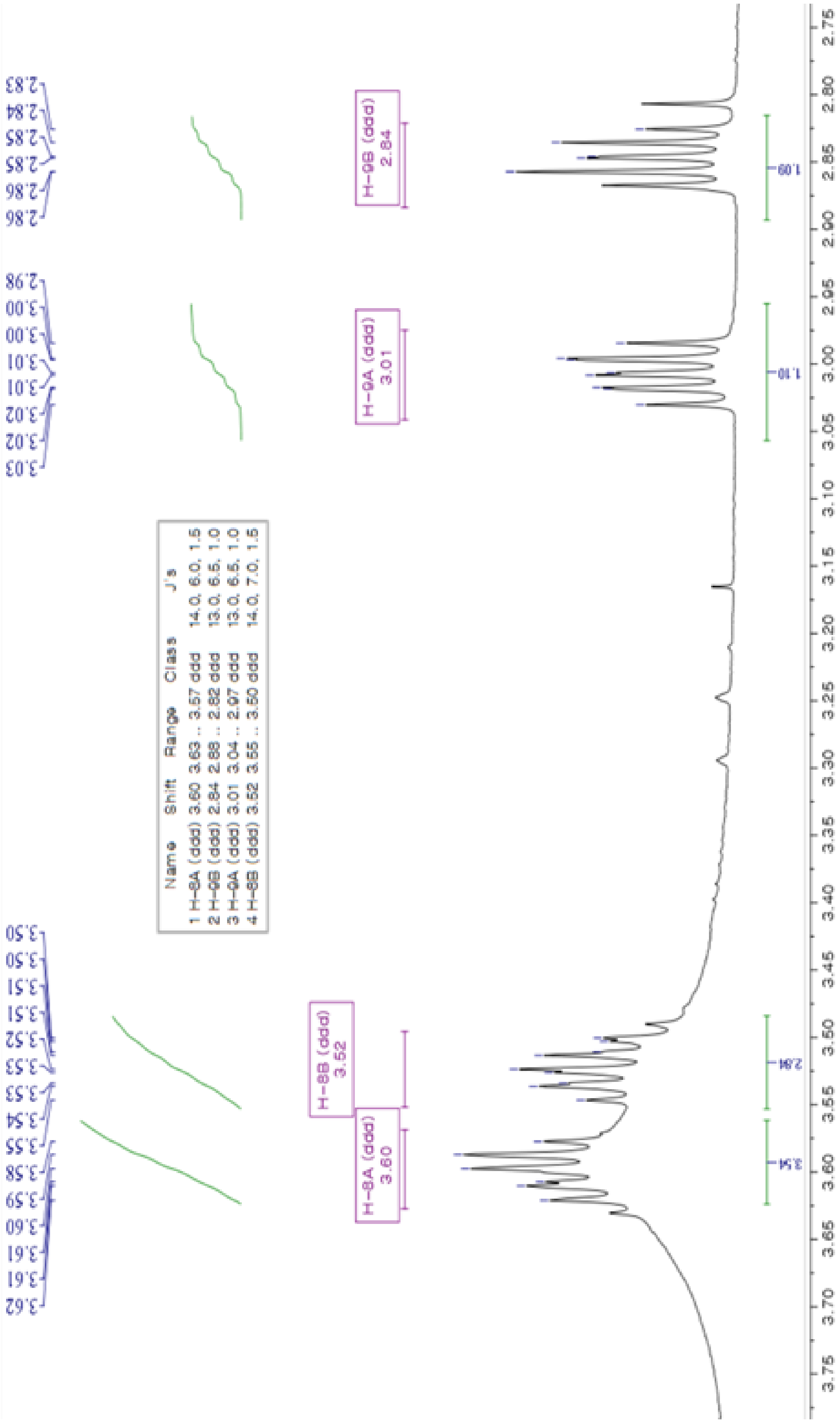

Figure S20. The ^{1}H NMR (600 MHz, DMSO-d_6) spectrum (δ_H 2.7 – 3.8 ppm) of **2**.
